# Microfabrication-Based Three-Dimensional (3-D) Extracellular Matrix Microenvironments for Cancer and Other Diseases

**DOI:** 10.3390/ijms19040935

**Published:** 2018-03-21

**Authors:** Kena Song, Zirui Wang, Ruchuan Liu, Guo Chen, Liyu Liu

**Affiliations:** College of Physics, Chongqing University, Chongqing 401331, China; kenasong@cqu.edu.cn (K.S.); cquphywzr@cqu.edu.cn (Z.W.); phyliurc@cqu.edu.cn (R.L.)

**Keywords:** microfabrication, extracellular matrix, cancer, metastasis

## Abstract

Exploring the complicated development of tumors and metastases needs a deep understanding of the physical and biological interactions between cancer cells and their surrounding microenvironments. One of the major challenges is the ability to mimic the complex 3-D tissue microenvironment that particularly influences cell proliferation, migration, invasion, and apoptosis in relation to the extracellular matrix (ECM). Traditional cell culture is unable to create 3-D cell scaffolds resembling tissue complexity and functions, and, in the past, many efforts were made to realize the goal of obtaining cell clusters in hydrogels. However, the available methods still lack a precise control of cell external microenvironments. Recently, the rapid development of microfabrication techniques, such as 3-D printing, microfluidics, and photochemistry, has offered great advantages in reconstructing 3-D controllable cancer cell microenvironments in vitro. Consequently, various biofunctionalized hydrogels have become the ideal candidates to help the researchers acquire some new insights into various diseases. Our review will discuss some important studies and the latest progress regarding the above approaches for the production of 3-D ECM structures for cancer and other diseases. Especially, we will focus on new discoveries regarding the impact of the ECM on different aspects of cancer metastasis, e.g., collective invasion, enhanced intravasation by stress and aligned collagen fibers, angiogenesis regulation, as well as on drug screening.

## 1. Introduction

The lethality of cancer lies in its ability to form metastases that accounts for about 90% of cancer deaths according to the available statistics [1,2]. The phenomenon of cancer metastasis has been investigated extensively in the last decade [3,4,5], and the neighboring microenvironment of cancer cells, i.e., the extracellular matrix (ECM), has been found to significantly impact tumor and metastasis development [6,7,8,9,10].

Cancer cells are not isolated, and their complicated cell–cell communications, development, metastases, and functions are always closely connected with the ECM microenvironment [11,12,13], e.g., tumor cells must break through the ECM, a critical step for cancer metastasis, to be able to reach the lymphatic or vascular system [14]. Therefore, an in-depth understanding of the interactions between cancer cells and ECM, from both physical and biological perspectives, is necessary to uncover the mechanism of cancer metastasis. This may also help to find potential therapeutic strategies to control malignant cancer. To achieve this goal, constructing a realistic in vitro cell culture system, particularly involving cell proliferation, migration, invasion, and apoptosis in relation to the ECM, becomes imperative.

In fact, the structure of the ECM in vivo is a complicated system, especially around a neoplastic tissue. The 3-D structure of the ECM in healthy, perilesional, and neoplastic tissues is different. The ECM in a healthy area shows a homogeneous distribution of structure, proteins, and glycoproteins, with collagen fibers intersecting to form a random network. Conversely, the ECM in perilesional and neoplastic areas shows a heterogeneous distribution of the structure, with a dense matrix, irregular shape, and asymmetric profile. The heterogeneous degree of glycoproteins distribution and the parallel degree of collagen fibrils become more obvious closer to the neoplastic tissue [15,16]. In addition, the degree of stiffness of the ECM is an important parameter related to the occurring lesions. The increased stiffness of perilesional areas may represent a new predictive marker of invasion [17].

Traditionally, cells have been cultured in Petri dishes that can only provide a two-dimensional (2-D) extracellular environment: cells can only attach to the surface of the medium and cannot form any 3-D scaffolds to mimic the real tissue complexity and functions [18,19,20]. Although some important discoveries have been made by using 2-D cancer cell culture systems, they are still insufficient for understanding the complex interactions between cancer cells and the ECM. Numerous studies have indicated that cell morphology, signaling patterns, and cellular functions are different in 3-D tissue microenvironments in vitro compared to 2-D Petri-dish systems [21,22], e.g., 2-D cell cultures do not fully support the recovery of the cellular phenotypes found in tissues in vivo [23]; also, when addressing drug toxicity effects, pharmacokinetic studies performed in 2-D polarized intestinal cells showed distinct features compared to those from toxicology screening tests conducted in a 3-D system composed of interconnected channels and chambers representative of distinct tissue types [24,25].

Realizing a 3-D tissue microenvironment similar to the one found in vivo, is one of the major challenges, but also the key factor to bridge the gap. In the past, many efforts were made to mimic the complex 3-D tissue microenvironment and particularly its influence on cell proliferation, migration, invasion, and apoptosis in relation to the ECM, for example by embedding cell clusters in a low-density Matrigel to form a lumina to mimic the in vivo epithelial layer [26], or by aggregating cells into 3-D spheroidal structures on a low-adhesion surface of a 2-D culture system [27]. However, a precise control of cell morphology (including size, density, and shape) is still lacking, and quantitatively adjustments of the external microenvironment, such as the medium and nutrition gradient, cannot be achieved [28].

In this review, we will discuss some commonly used, modern microfabrication techniques for the effective reconstruction of 3-D cell culture microenvironments in vitro that mimic real tissue structures or systems in vivo. On the basis of the available literature and the latest progress, we will elaborate on how these specifically designed 3-D cells–ECM microenvironments in vitro are applicable to the study of cancer, including metastasis, tumor angiogenesis, and drug screening, as well as of other diseases.

## 2. Microfabrication Techniques in the Effective Reconstruction of 3-D ECM Microenvironments In Vitro

Recently, the rapid development of microfabrication techniques, such as 3-D printing [29,30,31,32], microfluidics [33,34], photopolymerization, photochemistry, photoreaction [35,36,37,38,39,40], mold-based Diskoid In Geometrically Micropatterned ECM (DIGME) [41,42], as well as the corresponding cell culture approaches, have made it possible to precisely design and construct sophisticated controllable tissue models in vitro that mimic the 3-D architectures and physiological conditions present in vivo [28,43]. Consequently, various biofunctionalized hydrogels, such as collagen, fibronectin, and Matrigel, have become ideal candidates [12,44,45,46,47,48,49,50]. Furthermore, combined with advanced modern biomicroscopic tools, the direct and clear observation of cell morphology, motility, and other behaviors has become much easier, thereby helping the researchers acquire new insights into various diseases. Some commonly used methods and materials to engineer 3-D cancer or other disease models and the strengths and limitations of each technique are summarized in Table 1 and Table 2, respectively.

### 2.1. Microfabrication Techniques Contribute to the Construction of Special Structures, Such as Interface, Regions of Oriented Collagen Fibers, and Vascular Structures in the ECM

Metastasis usually involves the infiltration of cancer cells through aligned collagen fibers in the ECM and their subsequent penetration through the basement membrane to reach the blood or lymph vessels [14]. Moreover, characteristic changes in the orientation of the collagen fibers in the ECM are usually accompanied by the initiation and progression of tumors and are labeled as tumor-associated collagen signature. This collagen signature has served as an early diagnostic marker in various pathological processes related to cancer [60]. To mimic such processes, such as the ECM infiltration and collagen signature in in vitro cell culture systems, a successful reconstruction of the special structures of the ECM becomes significantly important. Microfabrication techniques seem to be ideal tools to realize this goal.

Zhu et al. [34] created a 3-D heterogeneous Matrigel structure, i.e., an ECM system with an interface inside it, to simulate the nonhomogeneous ECM microenvironment in vivo by curing two Matrigel sections of identical concentration at different times. Brin M. Gillette et al. [61] also fabricated a stable collagen fiber-mediated interface by micropatterning and gelling 3-D ECMs of flexible composition to mimic the nonhomogeneous and anisotropic properties of the native tissue.

**Figure 1:**
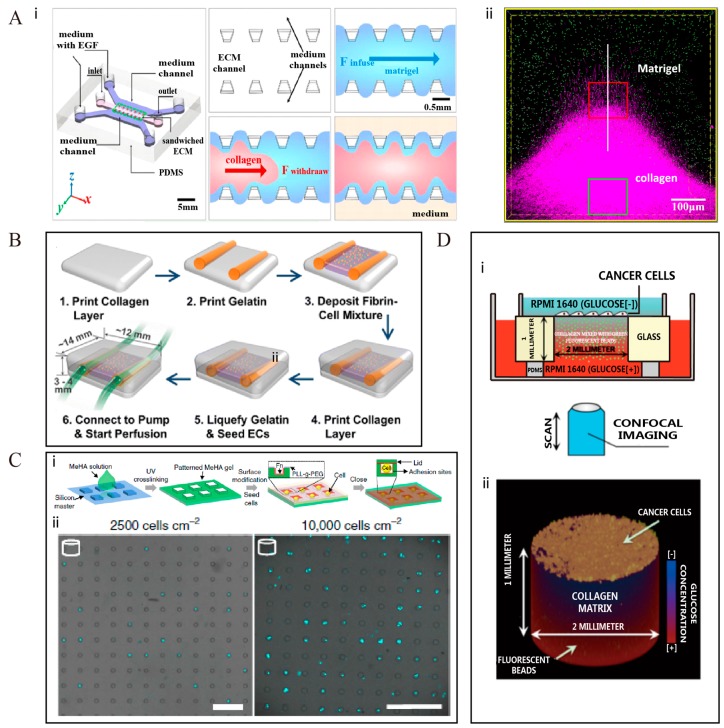
Diverse microfabrication techniques to recreate 3-D ECM microenvironments in vitro. (**A**) Microfluidics is used to build 3-D sandwiched ECM formed by collagen and Matrigel (i). An aligned collagen fiber zone is established at the surface (ii) [14]; (**B**) A multiscale vascular system with collagen as ECM is produced by using the 3-D bioprinting technology [63]; (**C**) Photochemistry is used to build 3-D patterned hydrogels as scaffolds with a range of different geometries (i). The size and shape of the cells is controlled inside microniche chambers (ii). Scale bar: 100 μm [23]; (**D**) A glucose gradient is established in the microfluidic chip, with collagen as the ECM. The system is used to study the trend of collective invasion of cancer cells. The cartoon of device is shown in (i), and the corresponding 3-D view is shown in (ii) [64]. All figures shown here are reproduced with permission from copyright owner.

Besides the realization of interfaces, microfabrication provides a new way to establish aligned collagen fiber structures. Liu et al. [14] created a 3-D sandwiched ECM microenvironment in a microfluidic chip with pillar-like inner structures by injecting collagen I in a fluid state into the Matrigel. Collagen I showed a specific orientation, perpendicular to the heterogeneous interface, induced by the internally developing strain during the solidification of the composite ECM, as shown in Figure 1A. Such aligned collagen fibers might resemble the highly oriented collagen fiber bundles found in cancer patients. Alexandra et al. [62] also varied the alignment of individual fibers and their network by using a microfabrication-based 3-D culture approach to investigate the dynamics of ECM alignment around tissues of defined geometry.

Other complex structures, such as in vascular or multicomponent systems, can also be reconstructed with modern microfabrication techniques. Lee et al. [63] developed a 3-D bioprinting platform, as illustrated in Figure 1B, to construct large-scale fluidic vascular channels with an adjacent capillary network within a 3-D hydrogel through a natural maturation process, as seen for angiogenic endothelial cells sprouting from a large channel edge and branching out into the collagen matrix. Their model can help to engineer desired 3-D vascular niches or thick vascularized tissues. Yong Da Sie et al. [38] adopted multiphoton excitation photochemistry to fabricate a complex 3-D multicomponent microstructure by inserting human fibronectin at specific locations on the 3-D scaffold formed by bovine serum albumin (BSA). Their model can be used to test the role of single factors in cell growth and metastasis.

### 2.2. Advanced Microfabrication Techniques Can Realize a Systematic and Quantitative Regulation of Cell Geometry, Such as Density, Volume, Size, Shape, and Location, in a Complex 3-D ECM Setting for Long Periods of Culture

Geometric cues are also considered to affect cellular functions, e.g., cell survival, self-renewal, and differentiation, in 3-D ECM microenvironments [23,65]. A precise control of cellular geometry in a complex 3-D scaffold, for long periods of culture, can be achieved effectively through the modern microfabrication techniques.

Lee et al. [66] fabricated a 3-D spheroid culture-based system with transparent and cell-repulsive polyacrylamide hydrogel, for testing nanoparticle toxicity. In their system, the sizes and shapes of the cells were accurately controlled by constraining the cells inside the pores. In 2011, Cheri Y. Li et al. [67] fabricated a 3-D cell-laden hydrogel microtissue with a photocurable hydrogel as the ECM. This system could control the cell size as well as the cell type. They made the microtissues functional by surface-encoding DNA. Through this system, they achieved multiple types of microtissues with multiplexed patterns. Several years later, Bao et al. [23] provided a more robust method to encapsulate single cells within 3-D matrix structures using the photopolymerization technique, for example in hydrogel microniches, which allows both cell adhesion and nutritional permeability, as shown in Figure 1C. These microniches were prism-shaped, with a controlled geometry of the bottom plane, and had precisely defined volumes, which could constrain the cell size and geometry in a systematic and quantitative manner. This device could also be easily combined with a confocal microscope to acquire images rapidly. 

### 2.3. Modern Microfabrication Techniques Can Help to Control Precisely the Concentration Gradient of the Medium, with Respect to Growth Factosr, Nutrients, and Drugs, in the ECM

Metastasis is an energy-consuming process, and cancer cell invasion cannot be achieved without a driving force, such as growth factors, nutrition, or drugs [66,68]. Microfabrication techniques can help us to setup a concentration gradient inside the ECM to further investigate the complex metastasis process in vitro. 

Liu et al. [64] designed a 3-D mesoscopic ecology by drilling a hole into a glass slide and filling it with 4.7 mg/ml collagen solution. A stable glucose gradient was established through the gel with low-glucose medium at the top and high-glucose medium at the bottom of the device, as demonstrated in Figure 1D. This device can help to test chemotaxis and trophotaxis of metastatic cells towards glucose or growth factors in a 3-D homogeneous ECM microenvironment. Ashley A. Jaeger et al. [56] also fabricated a 3-D bioreactor using Matrigel as the ECM. Their system could quantitatively control the oxygen gradient mimicking the tumor microenvironment, and could potentially be applied to study gas exchanges in cancer cell cultures.

Multiple concentration gradients can also be realized. Fan et al. [18] constructed a 3-D microfluidic system with an array of hollow microchambers in collagen I for cell culture, by employing the micromoulding and microfluidic techniques. This delicately designed microfluidic system could realize up to four controlled biochemical and drug gradients, simultaneously, through four independent microchannels encompassing the central collagen platform, and offered a robust platform for high-throughput drug and biochemical screening studies.

In conclusion, on the basis of the advanced microfabrication techniques, e.g., 3-D printing, microfluidics, and photochemistry, reconstructing a 3-D cells–ECM microenvironment in vitro, which perfectly mimics tissue morphology and functions in vivo, becomes an attainable goal. Especially, these techniques prove to be very useful in forming a special morphology, such as interface, oriented collagen fiber region, and vascular structures, or in quantitatively regulating cellular geometry and precisely controlling concentration gradients in the ECM.

## 3. New Discoveries about Cancer and Other Diseases, Based on 3-D ECM Systems In Vitro

Because of the rapid development of advanced microfabrication techniques, numerous novel discoveries, related to cancer and other diseases, have arisen in the last decade. In this section of the review, we will discuss some important studies and latest progress in the field of cancer and other diseases, based on 3-D in vitro cell–ECM microenvironments. We will especially focus on cancer metastasis—new discoveries of the impact of the ECM, e.g., collective invasion, intravasation enhancement by stress and aligned collagen fibers, angiogenesis regulation, as well as drug screening.

### 3.1. The Impact of Tumor Biotic Factors and Secretion on Normal Tissues and Cells via 3-D ECM Microenvironments

Cancer cells are reported to be able to affect normal tissues and cells by secreting biotic factors, which are transferred through the neighboring ECM. Therefore, a comprehensive understanding of the complicated interactions between cancer cells and normal tissues via a 3-D ECM would be of great benefit for a better control of malignant cancers.

**Figure 2:**
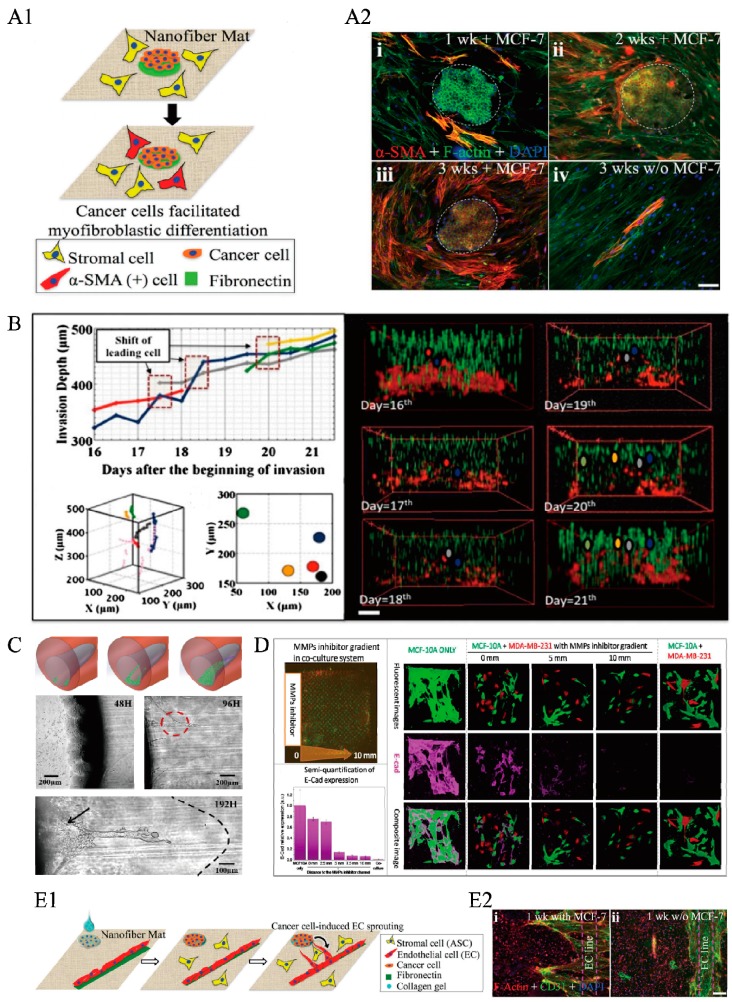
New discoveries about cancer and other diseases based on 3-D ECM systems in vitro. (**A1**) Spatiotemporal interactions between breast cancer cells and stromal cells are studied in a coculture system established by 3-D bioprinting technology. Cancer cells could recruit the stromal cells by differentiating into α-SMA positive cells, dashed line circle indicates the colony formed by cancer cells. (**A2**) Cancer cells (MCF-7) and stromal cells are co-cultured with high seeding density on the polycaprolactone nanofiber matrices. (i-iii) show the fluorescence images after co-cultured for 1 week to 3 weeks. The control seeding, without cancer cells, shows that only a small part of stromal cells became α-SMA-positive cells (iv) Scale bar: 100 μm [29]; (**B**) Using a 3D ECM of collagen, a glucose gradient is established for studying the collective invasion of cancer cells. Scale bar: 150 μm [64]; (**C**) A 3-D funnel-like Matrigel interface is created inside the ECM microenvironment. Through the complex and heterogeneous 3-D ECM microenvironment, ECM heterogeneity is proved to be an essential element in controlling collective cell invasive behaviors, red dashed circle indicates the network and connection of invading cells with other invading branches, and black arrow shows the cell plane. [34]; (**D**) A complex concentration gradient of specific biological molecules is built in a 3-D microfluidic system including microchamber arrays. Matrix Metalloproteinases (MMPs) produced by cancer cells are proven to play a dominant role in determining cellular behavior through the control of E-cad expression [18]; (**E**) Using a system made by bioprinting, cancer cells are shown to regulate angiogenesis through secretion. Cancer cells and endothelial cells are seeded far from each other (**E1**). Endothelial cells are found to sprout toward the cancer clusters. (**E2**) The fluorescence images of coculture of cancer cells and endothelial cells (i), and the control group of endothelial cells only (ii). Scale bar: 100 μm [29]. All figures shown here are reproduced with permission from copyright owner.

Eliza Li Shan Fong et al. [69] established a 3-D Ewing sarcoma model using an electrospinning apparatus ex vivo. They found that Ewing sarcoma cells cultured in porous 3-D electrospun poly(ε-caprolactone) exhibited remarkable differences in the expression pattern of the insulin-like growth factor-1 receptor/mammalian target of rapamycin pathway, and they were more resistant to traditional cytotoxic drugs compared to the cells grown in a 2-D monolayer culture system. Jia et al. [29] fabricated a 3-D ECM-like substrate with arbitrary micropatterns by combining inkjet printing and electrospinning. They found a close spatiotemporal interaction between breast cancer cells and stromal cells through transforming growth factor (TGF)-β1, which is secreted by breast cancer cells and can induce spatial differentiation of fibroblasts, as shown in Figure 2A1,A2.

### 3.2. The Collective Invasion of Cancer Cells into a Homogeneous ECM System Driven by Medium, Nutrients, and Growth Factor Gradients

During metastasis in vivo, cancer cells are commonly found to migrate to distant locations collectively. Extensive investigations have indicated that the intercellular and cell–ECM interactions have significant impacts on this collective invasion behavior.

Liu et al. [64] constructed a 3-D mesoscopic ecology to investigate the process of invasion of metastatic cells into an elastic medium in the presence of a glucose gradient. They showed that human breast cancer cells seeded on the top surface of the gel with low-glucose medium could invade into the 3-D collagen matrix cooperatively from the top to the bottom, where the glucose medium concentration was high. This collective invasion of cancer cells was found to be similar to that seen in the case of exchanging leaders in the invading front, as shown in Figure 2B. John Casey et al. [40] fabricated 3-D microwell arrays of defined geometry and dimension using photoreactive hydrogels to study cell migration out from cell aggregates over time. They concluded that spheroids within PEG 20 kDa microwells had the narrowest diameters as the cells tended to condense and cluster together.

Brendon M. Baker et al. [57] used the sacrificial channel template approach to fabricate another ECM structure, composed of collagen gel, which contained microfluidically ported microchannels. A stable diffusion gradient of soluble angiogenic growth factors dependent on the structure of the microfluidic network, was established. They showed that human umbilical vein endothelial cell (HUVEC) invasion and angiogenic sprouting occurred at the locations with the strongest gradients.

### 3.3. Intravasation of Cancer Cells into a Heterogeneous ECM Structure with the Help of Gradients, Stress, or Aligned Collagen Fibers

The intravasation of cancer cells into the lymphatic or vascular systems involves the key process of the cancer cells breaking through the basement membrane to get into the vessels. With modern technology, the important and puzzling question concerning tumor metastasis, i.e., how the heterogeneous ECM structures, such as aligned collagen or interface, influence cell infiltration and the subsequent break into the basement membrane during metastasis, is slowly getting answered.

In 2012, Ioannis K. Zervantonakis et al. [70] designed and constructed a 3-D tumor–endothelial intravasation microfluidic-based assay by interconnecting two independently addressable microchannels via a 3-D ECM hydrogel. Tumor and endothelial cells were seeded, respectively, in these two channels to mimic tumor cell invasion through 3-D ECM, in response to externally applied growth factor, such as epidermal growth factor (EGF), or biochemical gradients. Their experiments, aimed at addressing how tumor cells interact with normal epithelial cells, showed that breast carcinoma cells could invade through a HUVEC monolayer in the presence of macrophages, and the endothelial barrier impairment was associated with a higher number and faster dynamics of tumor–endothelial interactions. Several years later, Zhu et al. [34] used their system of 3-D heterogeneous ECM microenvironment in vitro to mimic the environment in vivo. Breast cancer cells were found to exhibit strikingly different invasive behaviors in homogenous and heterogeneous Matrigel with interior interfaces. As shown in Figure 2C, the interface in a heterogeneous ECM microenvironment guided cell invasion at an early stage, and the cells showed strong collective finger-like invasive behaviors. Liu et al. [14] used their 3-D ECM structure, as introduced in Section 2.1, to study cancer cell infiltration. They showed that the aligned collagen fibers were able to significantly enhance tumor metastatic potential, since metastatic breast cancer cells could accelerate their infiltration into rigid Matrigel by following the alignment direction. Epithelial–mesenchymal transition (EMT) is also a prominent signal of deterioration of tumors. Once a cancer cell undergoes a phenotypic change through EMT, it will obtain greater migratory and invasive ability, directing cells to penetrate into the vessels [71]. Sharmistha Saha et al. [72] combined the technology of electrospun and microfabrication to create fibrous scaffolds with random or aligned fiber orientation mimicking the complex ECM microenvironment in tumors. They found that the aligned fiber scaffold can guide EMT in cancer cells accompanied by the upregulation of TGF β-1.

### 3.4. Biophysical and Mechanical Properties of the ECM, such as Geometry, Density, Stiffness, Contractility, and Crosslinking of Collagen Fibers, Influence Tumor Angiogenesis Both Directly and Indirectly

Recently, ECM stiffness has progressively become a significant mechanical cue that precedes disease and drives its pathological progression by altering cellular behavior or influencing tumor angiogenesis [73]. Abnormal vessel growth and function are usually the hallmarks of cancer or inflammatory diseases and contribute to disease progression [74]. Tumor cells and tissues need to establish a blood supply, primarily through angiogenesis, from a pre-existing vascular network, to satisfy their increasing demand for nutrients and oxygen to perform metabolic functions, similar to the normal tissues [74,75]. Angiogenic programming of tumor tissues is a multidimensional process, regulated by cancer cells, stromal cells, their bioactive products, as well as ECM microenvironments [76].A few years ago, Mason et al. [77] took advantage of the tunable mechanical properties of collagen-based scaffolds to investigate the effects of matrix stiffness on 3-D microenvironments. They observed a significant increase in cellular spreading and extension with increasing matrix stiffness, and these effects lasted throughout the entire course of the cell culture period. Recently, Jia et al. [29] showed cancer-regulated angiogenesis by factors secreted from the cancer cells, which guided the sprouting of endothelial cells toward cancer clusters, by using their 3-D ECM structure with arbitrary micropatterns, as shown in Figure 2E1,E2.

### 3.5. 3-D Microenvironments for Drug Screening

Conventionally, pharmacokinetic studies are performed in rodents or in 2-D polarized intestinal cells before clinical administration, nevertheless, several compounds have still proven toxic to humans, despite such testing [9]. Advanced microfabrication techniques have proven their suitability in creating 3-D cell–ECM microenvironments in vitro that can be perfectly used for screening drugs.

Lee et al. [66] showed that the toxicity of CdTe and Au was significantly reduced in their system with a 3-D microenvironment, compared to that in 2-D cultures. In 2017, Fan et al. [18] used their complex gradient microfluidic system, which allowed incorporating multiple chemical, temporal, and spatial gradients within a 3-D micropatterned collagen scaffold in vitro, to test the role of matrix metalloproteinases (MMPs) in inhibiting cancer cells aggregation. As shown in Figure 2D, MMPs inhibitors were found to depress the abilities of the cancer cells to disrupt the structure of the surrounding lumen-like cell clusters; the down regulation of E-cad expression, due to the MMPs produced by invasive breast cancer cells, played a dominant role in determining the cellular behavior.

### 3.6. Other Diseases (Wound Healing, Clonal Acinar Development, Differentiation of Embryoid Bodies, Neurodevelopmental Processes, Cartilage Defects) Related to 3-D Cells–ECM Microenvironments

Besides cancer metastasis, 3-D cell–ECM microenvironments fabricated by modern microfabrication techniques can also be applied in the study of many other diseases or biomedical processes, including, but not limited to, wound healing, clonal acinar development, differentiation of embryoid bodies, neurodevelopmental processes, and so on.

The idea of the 3-D bioprinting of skin cells was considered adequate for transitioning the technology to clinical settings. For example, Skardal, A. et al. [78] used the bioprinting technology to deposit two layers of a fibrin–collagen gel containing endothelial cells to treat full-thickness skin wounds in mice.

In 2015, Dolega et al. [79] constructed a 3-D cell culture system based on a flow-focusing microfluidic chip that encapsulates cells in Matrigel microbeads. Each individual microbead acts as a single-cell culture compartment, generating one acinus per bead, on an average. They used this 3-D cell culture system to investigate acinar development, by tracing the microbead under temporally and spatially controlled conditions. They found that acinar development proceeds through clonal growth from single cells, which are self-sufficient to differentiate in a Matrigel environment. 

In studying the differentiation of embryoid bodies, Fung et al. [80] presented a 3-D microfluidic platform, using biocompatible materials, based on a Y-channel device with two inlets for two different culturing media, to study the differentiation of embryoid bodies by in vitro aggregation of embryonic stem cells. They found that the differentiation of the same embryonic stem cells into different specialized cell lineages can be achieved by applying the microfluidic technology.

In the treatment of cartilage defects, the differentiation of stem cells into chondrocytes is an important cue [81]. Many physical and mechanical characteristics of the ECM, such as hypoxia, stiffness, topography, and pore size, are closely related to the interdifferentiation process of chondrocytes and stem cells [82]. Kuan-Han Wu et al. [81] used gelatin as ECM in a microfabrication process to create a scaffold with different sizes of microbubbles and pores. They found that the size of microbubbles and pores can impact chondrogenesis, and a larger pore size is required for inducing mature chondrogenesis. Xiaobin Huang et al. [83] chose pH-degradable Poly(vinyl alcohol) (PVA) hydrogel as an ECM scaffold to study the effects of oxygen tension on chondrogenesis. They found that the degree of hypoxia affects the expression of many cytokines (e.g., MMP13, ALPL, COL10A1, ELISA), which has a further influence on chondrogenesis. For example, hypoxia in 2.5% O_2_ would promote cartilage matrix production.

Recently, Lancaster et al. [84] developed a 3-D cerebral organoid culture system for deriving brain tissues to study human neurodevelopmental processes in vitro. This method recapitulated not only the fundamental mechanisms of mammalian neurodevelopment, but also displayed a wide range of characteristics of human brain development.

## 4. Conclusions

The significant progress of microfabrication in the last decade has advanced our understanding of cancer and other diseases. In this review, we have discussed some important findings and latest developments of the above approaches with respect to cancer and other diseases in 3-D ECM scaffolds. We stress that these 3-D ECM microenvironments are closely related to the process of cancer metastasis that presents multiple aspects, such as collective invasion and intravasation, tumor angiogenesis regulation, as well as drug screening.

## Figures and Tables

**Table 1:** Methods and materials used to engineer 3-D cancer or other disease models.

Technique	Method	Material
3-D bioprinting	BIP (bioink printing) [29] Extrusion printing [51] Laser-assisted bioprinting [37,38] Microvalve printing [52,53,54,55]	Hydrogel Biomolecular ink [29] Matrigel alginate [51] Chitosan [52] Gelatin [52] Alginate [54] Fibrinogen [55]
microfluidics	Soft lithography [33,35,36,56,57,58,59]	Matrigel [58] Collagen [18,33,35,57] Gelatin [57] Calcium alginate [58,59]
photochemistry	FLDW (femtosecond laser direct writing) [38] DMD-PP process(digital micromirror device-based projection printing) [37] UV-light exporsure [39]	BSA [38] Fibronectin [38] Polyethylene glycol (PEG) [37] Poly(ethylene glycol) diacrylate (PEGDA) [37] Methacrylamide-modified gelatin or hydrogel [39]

**Table 2:** Strengths and limitations of each technique.

Technique	Strength	Limitation
3-D bioprinting	Flexible	Unable to achieve accuracy at less than 50 μm (the highest accuracy is inkjet printing in publishing). The temperature is not easy to control precisely. High cost.
microfluidics	High precision Stable	Structures are limited, e.g., multilayers, lumen.
photochemistry	Curing fast High precision	Limited by optical characteristics. Must be combined with other technologies, e.g., 3-D printing, to achieve high accuracy and flexible structure.

## Abbreviations

3-D	Three dimensional
2-D	Two dimensional
ECM	Extracellular matrix
BSA	Bovine serum albumin
TGF	Transforming growth factor
HUVEC	Human umbilical vein endothelial cells
MMPs	Matrix metalloproteinases
EGF	Epidermal growth factor
DIGME	Diskoid In Geometrically Micropatterned ECM
EMT	Epithelial-mesenchymal transition
PEG	Polyethylene glycol
PEGDA	Poly(ethylene glycol) diacrylate
DMD-PP	Digital micromirror device-based projection printing
FLDW	Femtosecond laser direct writing
MMPs	Matrix Metalloproteinases
PVA	Poly(vinyl alcohol)
